# Phylogenetic Distribution of Polysaccharide-Degrading Enzymes in Marine Bacteria

**DOI:** 10.3389/fmicb.2021.658620

**Published:** 2021-03-18

**Authors:** Zhong-Zhi Sun, Bo-Wen Ji, Ning Zheng, Meng Wang, Ye Cao, Lu Wan, Yi-Song Li, Jin-Cheng Rong, Hai-Lun He, Xiu-Lan Chen, Yu-Zhong Zhang, Bin-Bin Xie

**Affiliations:** ^1^Microbial Technology Institute and State Key Laboratory of Microbial Technology, Shandong University, Qingdao, China; ^2^School of Life Sciences, Central South University, Changsha, China; ^3^College of Marine Life Sciences and Frontiers Science Center for Deep Ocean Multispheres and Earth System, Ocean University of China, Qingdao, China; ^4^Marine Biotechnology Research Center, State Key Laboratory of Microbial Technology, Shandong University, Qingdao, China

**Keywords:** marine bacteria, polysaccharide-degrading enzymes, phylogeny, ecological differentiation, genomics, carbohydrate active enzymes

## Abstract

Deconstruction is an essential step of conversion of polysaccharides, and polysaccharide-degrading enzymes play a key role in this process. Although there is recent progress in the identification of these enzymes, the diversity and phylogenetic distribution of these enzymes in marine microorganisms remain largely unknown, hindering our understanding of the ecological roles of marine microorganisms in the ocean carbon cycle. Here, we studied the phylogenetic distribution of nine types of polysaccharide-degrading enzymes in marine bacterial genomes. First, we manually compiled a reference sequence database containing 961 experimentally verified enzymes. With this reference database, we annotated 9,335 enzyme sequences from 2,182 high-quality marine bacterial genomes, revealing extended distribution for six enzymes at the phylum level and for all nine enzymes at lower taxonomic levels. Next, phylogenetic analyses revealed intra-clade diversity in the encoding potentials and phylogenetic conservation of a few enzymes at the genus level. Lastly, our analyses revealed correlations between enzymes, with alginate lyases demonstrating the most extensive correlations with others. Intriguingly, chitinases showed negative correlations with cellulases, alginate lyases, and agarases in a few genera. This result suggested that intra-genus lifestyle differentiation occurred many times in marine bacteria and that the utilization of polysaccharides may act as an important driver in the recent ecological differentiation of a few lineages. This study expanded our knowledge of the phylogenetic distribution of polysaccharide enzymes and provided insights into the ecological differentiation of marine bacteria.

## Introduction

Polysaccharides are high molecular weight organic substances highly abundant in nature, and their degradation is a key step in the carbon cycle. Deconstruction of the polysaccharides into oligosaccharides and mono-saccharides is the initial step of polysaccharide degradation (Mühlenbruch et al., 2018). Microbial genomes encode various types of polysaccharide-degrading enzymes (PDEs) (Flint et al., 2008; Talamantes et al., 2016). In many microbial genomes, genes for these enzymes as well as other functionally related enzymes and transporters may form clusters, called polysaccharide utilization loci (PUL), to enhance polysaccharide utilization (Grondin et al., 2017). Studies of degrading enzymes toward polysaccharides such as cellulose, chitin, and xylan in microorganisms have revealed broad distribution on the phylogenetic tree (Berlemont and Martiny, 2013, 2015; Nguyen et al., 2018). However, the phylogenetic diversity of degrading enzymes toward marine polysaccharides and encoding potentials for these enzymes in marine microorganisms remain largely unknown.

There are various kinds of polysaccharides in the marine environments. These polysaccharides include those also rich in the terrestrial environments, such as cellulose and chitin (Rinaudo, 2006; López-Mondéjar et al., 2016) and those absent in the terrestrial environments, for example, agar and carrageenan from the red algae (Xu et al., 2017; Ghanbarzadeh et al., 2018). These polysaccharides also include those present in the terrestrial environments but with a low amount. For example, the alginate is a kind of rich polysaccharide produced by brown algae (Michel et al., 2010). Terrestrial bacterial strains from two genera *Azotobacter* and *Pseudomonas* can produce derivatives of alginates, called bacterial alginates (Rehm and Valla, 1997). Marine polysaccharide-degrading enzymes are found from marine animals and bacteria (Michel and Czjzek, 2013; Ojima et al., 2018; Sun et al., 2020). Recently, there are increasing interests in the carbohydrate active enzymes from marine microorganisms (Hehemann et al., 2010; Klippel et al., 2014; Lin et al., 2018). New PDEs and polysaccharide utilization loci have been reported from marine bacteria (Kabisch et al., 2014; Larsbrink et al., 2016; Reisky et al., 2019).

The aim of this study is twofolds. First, this study will investigate the phylogenetic distribution of PDEs in marine bacteria to gain insights into the metabolism and ecological roles of various groups of marine bacteria. Second, this study will provide a collection of manually curated reference sequences and carefully annotated sequences of PDEs that can be used as the reference for the future genomic and metagenomic studies.

## Materials and Methods

### Reference Sequences

An initial reference sequence catalog for 10 types of PDEs, including 21 glycoside hydrolase (GH) families and 12 polysaccharide lyase (PL) families, was created by obtaining sequences noted as “Characterized” in the CAZyme database (Lombard et al., 2013). Then, a literature search was performed, and only sequences whose activities were supported by enzymatic assays were included in the reference sequence database. Besides, the reference sequence database also included 31 sequences whose activities were supported by literature, although their accessions were not included in the CAZyme database at the time of collecting reference sequences. The environmental source information (marine, other source or unknown) of the references was also compiled based on the literature. Taxonomic ranks of the source organisms were obtained from the NCBI Taxonomy database (Federhen, 2011).

### Marine Bacterial Genomes

All genome sequences (173,530) available at the NCBI RefSeq database (version November 2019) were downloaded. Based on the BioSample ID, the “isolation source” for each genome was retrieved from the NCBI BioSample database (Barrett et al., 2011). An experience-based marine-related word list was compiled to identify bacterial genomes from the marine environments (Supplementary Table S1), and as a result, 2,730 genomes were chosen as marine bacterial genomes. Next, the qualities of these genomes were evaluated to include only those with (1) N50 ≥ 50,000 bp, (2) completeness ≥ 95% and contamination ≤ 5% as calculated with checkM (Parks et al., 2015), and (3) all 31 marker genes detected with AMPHORA2 (Wu and Scott, 2012). Besides, redundant assemblies were removed so that only one assembly was kept for each strain. Finally, 2,182 genomes were obtained for further annotation and phylogenetic analyses. Taxonomic ranks of the source organisms were obtained from the NCBI Taxonomy database (Federhen, 2011).

### Annotation

A pipeline combining BLAST and HMMER search was developed to achieve reliable annotation. First, all reference sequences were searched against dbCAN HMMdb v8 (Zhang et al., 2018) using HMMER (V3.1b2, hmmscan-tblout) to obtain the position of the catalytic domain. Second, all protein sequences from each genome were used as query to search the reference database using BLAST (V2.7.1 +, blastp -evalue 0.001-outfmt “6 qseqid stitle pident qcovs qlen slen length mismatch gapopen qstart qend sstart send evalue bitscore”) and the proteins with local identity ≥ 30% at the matching region were kept as candidate enzymes for further filtering. Then, each candidate was scanned for catalytic domains using HMMER against dbCAN HMMdb v8. A candidate enzyme sequence was included in our final data set if (1) it had the same catalytic domain as its best-matched reference sequence as revealed by the above BLAST search, and (2) ≥80% of the catalytic domains for both the candidate and the reference fell in the BLAST matching region. Finally, each sequence in the final data set was annotated as the same enzyme as its best-matched reference sequence. Different identity and coverage thresholds were also tested.

### Phylogenetic Analysis

To evaluate the phylogenetic distribution, the 16S rRNA gene was extracted from each genome, and as a result, 16S rRNA gene sequences (length > 1,400 bp) for 1,819 genomes were obtained. Sequences were aligned with MAFFT (v7.450) with default parameters (Katoh and Standley, 2013), and then, a maximum likelihood tree was reconstructed using IQ-TREE (v1.6.2) with parameters (-m GTR+F+R5 -bb 1000) after an auto model selection (Nguyen et al., 2015).

In order to include all genomes in the phylogenetic analysis, the core genes were also used to reconstruct the phylogeny. AMPHORA2 was used to extract 31 marker genes from each genome (Wu and Scott, 2012), and these genes were used to reconstruct the core gene tree. Sequences of each gene were aligned with MAFFT (v7.450) using default parameters (Katoh and Standley, 2013) and then processed with trimAl (v1.4, parameter: -gt 1) to remove poorly aligned regions (Capella-Gutierrez et al., 2009). The obtained alignments were connected, and then, a core gene phylogenetic tree was reconstructed using IQ-TREE (v1.6.2) with parameters: -m LG+F+G -bb 1000 (Nguyen et al., 2015).

Both the phylogenetic tree and the distribution of the enzymes were displayed with the iTOL server (Letunic and Bork, 2019).

### Phylogenetic Conservation Analysis

The consenTRAIT algorithm (Martiny et al., 2013) was developed to identify the clades with more than 90% of strains sharing a specific trait and to calculate the average trait depth (τ_*D*_) as the average phylogenetic depths (i.e., the summed branch length from each leaf node to the root of the clade) of all identified clades. Here, the algorithm was used to evaluate the phylogenetic conservation of each type of enzyme. This algorithm was developed to work with the 16S rRNA tree, and as a result, the τ_*D*_ value can be easily inter-converted with the 16S rRNA sequence identity between members in the identified clades. The 16S rRNA identity can be calculated as the τ_*D*_ value multiplied by two and then subtracted from 1. The τ_*D*_ values 0, 0.01, 0.02, 0.03, 0.04, and 0.05 indicated average 16S rRNA identities 100%, 98%, 96%, 94%, 92%, and 90%, respectively. Since not all genomes in our data set contained 16S rRNA gene sequences, the core gene tree was also used in this study to incorporate all genomes. The τ_*D*_ values were calculated based on the 16S rRNA tree, and the core gene tree could not be compared directly due to the different tree lengths.

### Correlation Analysis

To evaluate the potential correlation of distribution between different enzymes, a vector containing the absence/presence information (absence: 0 and presence: 1) in each strain was calculated for each enzyme. Then, the Pearson correlation coefficients between enzymes were calculated based on these vectors using the “cor.test” function from “stats” package in “R”, and the Jaccard distances between enzymes were calculated using the “distance” function from “philentropy” package in “R”.

## Results

### Collection of Reference Sequences

**FIGURE 1:**
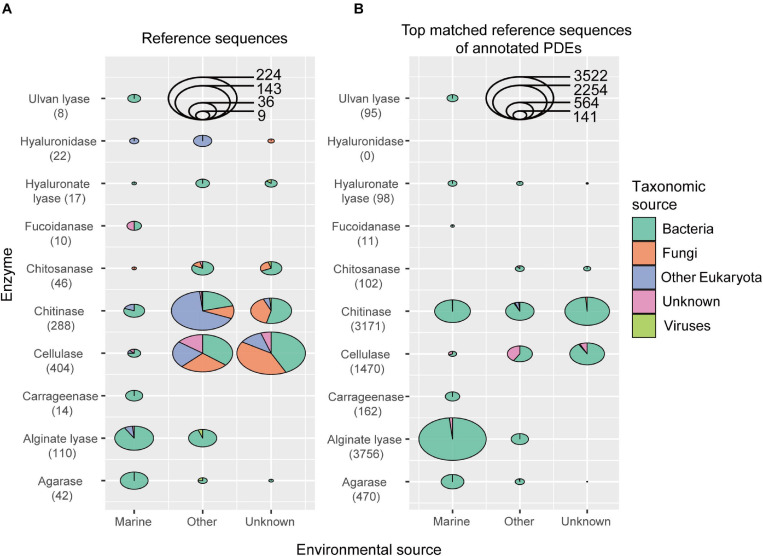
The environmental and taxonomic sources of sequences in the reference data set **(A)** and the top matched reference sequences of annotated PDEs **(B)**. The number of enzyme sequences was shown in parentheses.

A total of 10 types of PDEs for polysaccharides present in the marine environments were collected, including cellulases (substrate cellulose), chitinases (chitin), chitosanases (chitosan), agarases (agarose), carrageenases (carrageen), alginate lyases (alginate), focoidanases (focoidan), hyaluronate lyases (hyaluronate), hyaluronidases (hyaluronate), and ulvan lyases (ulvan). As a result, the reference collection included 961 sequences belonging to 33 CAZy families (21 GH and 12 PL families) (Supplementary Table S2). These sequences came from different organisms, including bacteria, fungi, other eukaryotes, and viruses, and from different environments, including land and ocean (Figure 1A and Supplementary Table S2). Among the enzymes included in the reference database, cellulases, chitinases, and alginate lyases were the most abundant. All sequences in this reference database were supported by enzymatic assays in the literature.

### Annotated PDEs in Marine Bacterial Genomes

A total of 2,182 non-redundant marine bacterial genomes were obtained from the NCBI RefSeq database and subject to further PDE annotation (Supplementary Table S3). They were from 481 genera of 19 phyla (divisions), and approximately a half were from *Gammaproteobacteria*.

Here we developed an annotation procedure to obtain reliable predictions. Briefly, the predicted protein must match its reference sequence at the catalytic domain with a relatively high coverage and local identity (see Materials and Methods section for detail; choice of coverage and identity cutoffs was discussed in Discussion section). With the above reference sequences and the procedure, we were able to predict 9,335 PDE genes from the above 2,182 genomes, with 1,370 genomes each containing at least one PDE gene (Supplementary Table S4). Since hyaluronidases were not annotated in the marine bacterial genome data set (Figure 1B), only nine types of PDEs were included in the following analyses.

The top-matched reference sequence was picked for each annotated PDE, and the taxonomic groups and environmental sources for these sequences were analyzed (Figure 1B). It was noted that, for three (chitinase, cellulase and alginate lyase) out of the nine types of PDEs annotated, there were sequences annotated with non-bacterial sequences as the reference, suggesting possible inter-kingdom gene transfer in the evolution of PDEs. It was also noted that, a high number of sequences were annotated with non-marine sequences as the reference, suggesting possible gene transfer between marine and other environments.

### Distribution in Different Taxa

**FIGURE 2:**
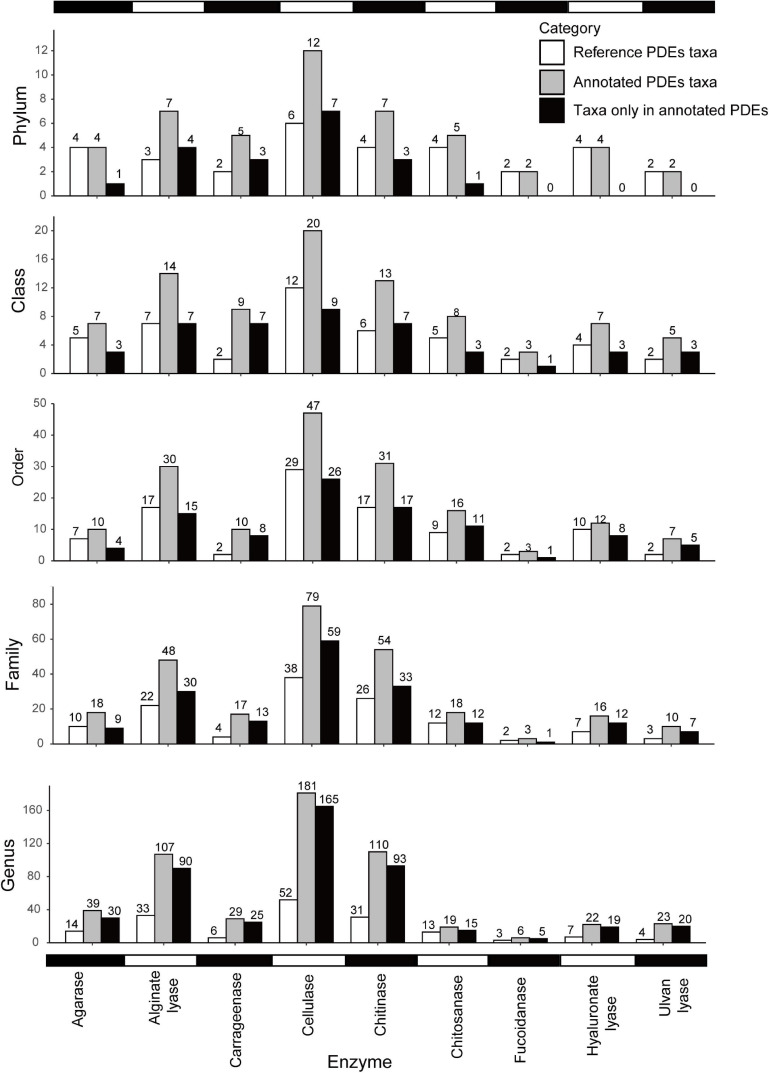
Distribution of PDEs at different taxonomic levels (phylum, class, order, family, and genus). Note that genomic annotation greatly expanded the distribution of PDEs. White, PDE reference sequences; gray, annotated PDEs; black, taxa only found in annotated PDEs.

**FIGURE 3:**
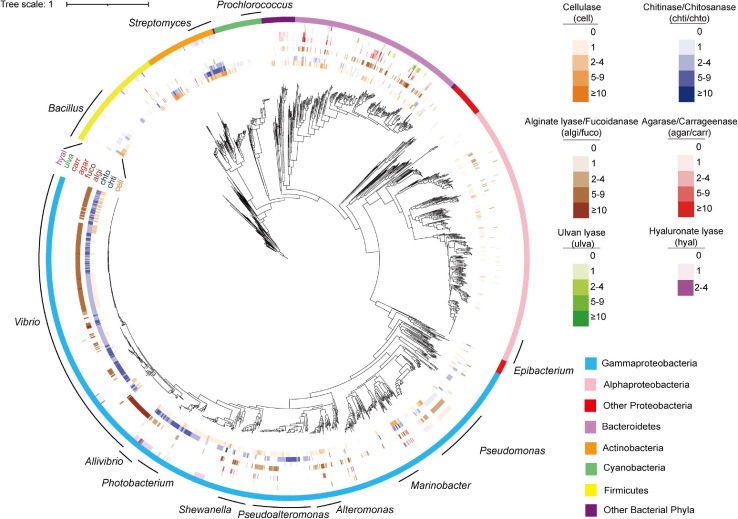
Phylogenetic distribution of PDEs among marine bacterial genomes. The tree was reconstructed based on 31 core genes using IQ-TREE. The figure was prepared with the help of the iTOL server. Enzymes were shown in outer circles as heatmap based on annotated gene number in each genome. The 12 genera subject to distribution analyses at the genus level were indicated with curves.

As shown in Figures 2, 3, different enzymes had different distributions. Cellulases showed the broadest distribution among the enzymes studied. They were found in 181 genera from 12 phyla (divisions). Chitinases and alginate lyases were also widely distributed, and they were found in 110 genera (seven phyla) and 107 genera (seven phyla), respectively. Fucoidanases had the narrowest distribution and were only found in six genera from two phyla (*Psychromonas* in *Gammaproteobacteria* and five other genera in *Bacteroidetes*). The uneven distribution of different enzymes may be a result of the different abundances of these families in the reference data set. It might also suggest different utilization potentials of marine bacteria toward different polysaccharides.

Compared with the reference data set, our annotation expanded the distribution of six enzymes at the phylum level and the distribution of all nine enzymes at lower levels (Figure 2). For example, cellulases were found in 12 phyla of marine bacteria but only found in six phyla in the reference data set. Alginate lyases were found in seven phyla in the final data set, compared to only three phyla in the reference data set. At the genus level, the distribution had been increased to 1.46 folds (chitosanases) to 4.83 folds (carrageenases) of that in the reference data set.

**FIGURE 4:**
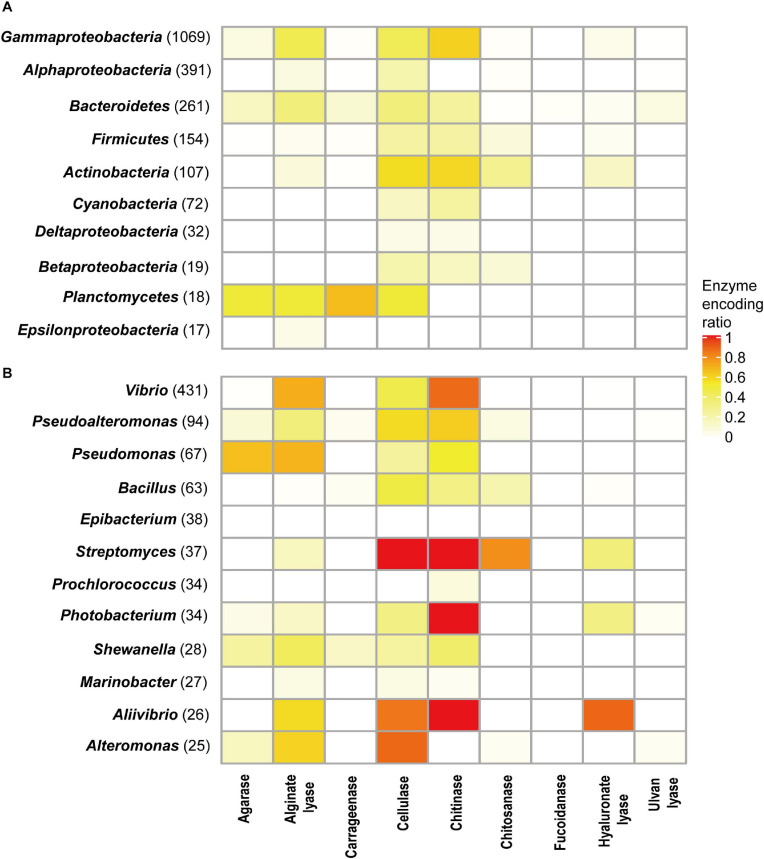
PDE encoding potentials by different taxa at the phylum **(A)** and genus **(B)** levels. The number of genomes available in each group was shown in parentheses.

Different phyla had different encoding abilities for PDEs (Figure 4A). All nine types of enzymes were found in *Proteobacteria* (*n* = 1,532) and *Bacteroidetes* (*n* = 261). In *Thermotogae* genomes (*n* = 8), only cellulases were found. In *Cyanobacteria* (*n* = 72), only cellulases and chitinases were found.

The ratios of trait-positive strains in a taxon were calculated for taxa with a relatively high number of genomes. At the phylum level, the highest ratios (58.9% for chitinases and 57.0% for cellulases) were observed in *Actinobacteria* (genome number = 107). Higher ratios were observed at lower taxonomic ranks. As shown in Figure 4B, analyses revealed a number of genera (genome number ≥ 25) that encoded a specific type of enzymes at high ratios. For example, 87.2% of *Vibrio* genomes (total number 431) encoded chitinases, and 70.5% of *Vibrio* genomes encoded alginate lyases. All *Streptomyces* genomes (*n* = 37) encoded cellulases and chitinases, and 78.4% of them encoded chitosanases. Meanwhile, it was also noted that ratios were low for a few enzymes encoded by a large number of genera (Figure 4B). Such heterogeneity in encoding abilities suggested diversity in the metabolism and ecological functions of bacteria from these genera.

### Phylogenetic Distribution

We further analyzed the distribution of different enzymes on the phylogenetic tree with the consenTRAIT algorithm. This algorithm could identify phylogenetic clusters whose members conservatively demonstrate the same trait and also estimate the average phylogenetic depth of all identified clusters on the tree (the mean trait depth, τ_*D*_). The phylogenetic distribution of each enzyme was first studied based on the 16S rRNA gene tree (containing 1,819 genomes). The mean trait depth for fucoidanase should be biased since all detected positive clusters were singletons (Table 1). For other enzymes, the mean trait depth ranged from 0.0144 (chitosanases) to 0.0281 (chitinases), which corresponded to 16S rRNA identities from 97.1% (chitosanases) to 94.4% (chitinases). Agarases (0.0235), cellulases (0.0226), and alginate lyases (0.0212) showed bigger mean trait depths than carrageenases (0.0197), hyaluronate lyases (0.0197), and ulvan lyases (0.0193). In order to utilize information of all available marine bacterial genomes, the phylogenetic distribution was also studied based on the core gene tree (Table 1), which was reconstructed based on 31 core genes as detected by AMPHORA2. Similarly, chitinases showed the biggest mean trait depth (0.0444). Cellulases (0.0370), agarases (0.0320), and alginate lyases (0.0312) also showed relatively big mean trait depths. Differently, hyaluronate lyases showed the smallest mean trait depth (0.0177). Chitosanases, which had the smallest mean trait depth with the 16S rRNA tree, showed the second biggest mean trait depth (0.0384) with the core gene tree.

**TABLE 1:** Phylogenetic conservation of enzyme-positive genotypes.

**Enzyme**	**16S rRNA gene tree**				**Core gene tree**			
	**Enzyme-positive genotypes**	**Singletons**	**Mean clade size**	**τ_*D*_**	**Enzyme-positive genotypes**	**Singletons**	**Mean clade size**	**τ_*D*_**
Agarase	114	36.80%	1.93	0.0235380	138	30.43%	2.28	0.0319636
Alginate lyase	524	25.80%	2.97	0.0211616	640	21.72%	3.40	0.0312082
Carrageenase	56	50.00%	1.68	0.0197299	64	46.88%	1.60	0.0262650
Cellulase	632	33.50%	2.31	0.0226166	780	25.00%	2.85	0.0369611
Chitinase	644	13.50%	4.61	0.0280873	839	10.13%	5.94	0.0444206
Chitosanase	60	41.70%	1.76	0.0144015	70	31.43%	2.48	0.0383783
Fucoidanases	5	100.00%	1	0.0507775	6	100.00%	1.00	0.0408922
Hyaluronate lyase	60	30.00%	2.38	0.0196562	86	40.70%	1.95	0.0177127
Ulvan lyase	26	73.10%	1.18	0.0193213	30	63.33%	1.25	0.0203285

As shown above, although there were differences in the ranking of the mean trait depth between the two trees, the ranking was generally (at least partially) consistent with the number and distribution of annotated PDEs. Furthermore, the mean clade size showed a better correlation with the number and distribution of predicted PDEs than the mean trait depth. As for the broadness of distribution (at the genus level), cellulases, chitinases, and alignate lyases ranked first, second, and third among the enzymes studied, respectively (Figure 2, bottom panel). They ranked fourth, first, second, respectively, in the mean clade size with the 16S rRNA tree and ranked third, first, second, respectively, with the core gene tree (Table 1). This result is reasonable since the mean clade size represented the number of nodes (strains) contained in the clade. Differently, the mean trait depth described the average branch length from the leaf nodes in the clade to the clade root.

### Correlated Distributions Between Enzymes

**FIGURE 5:**
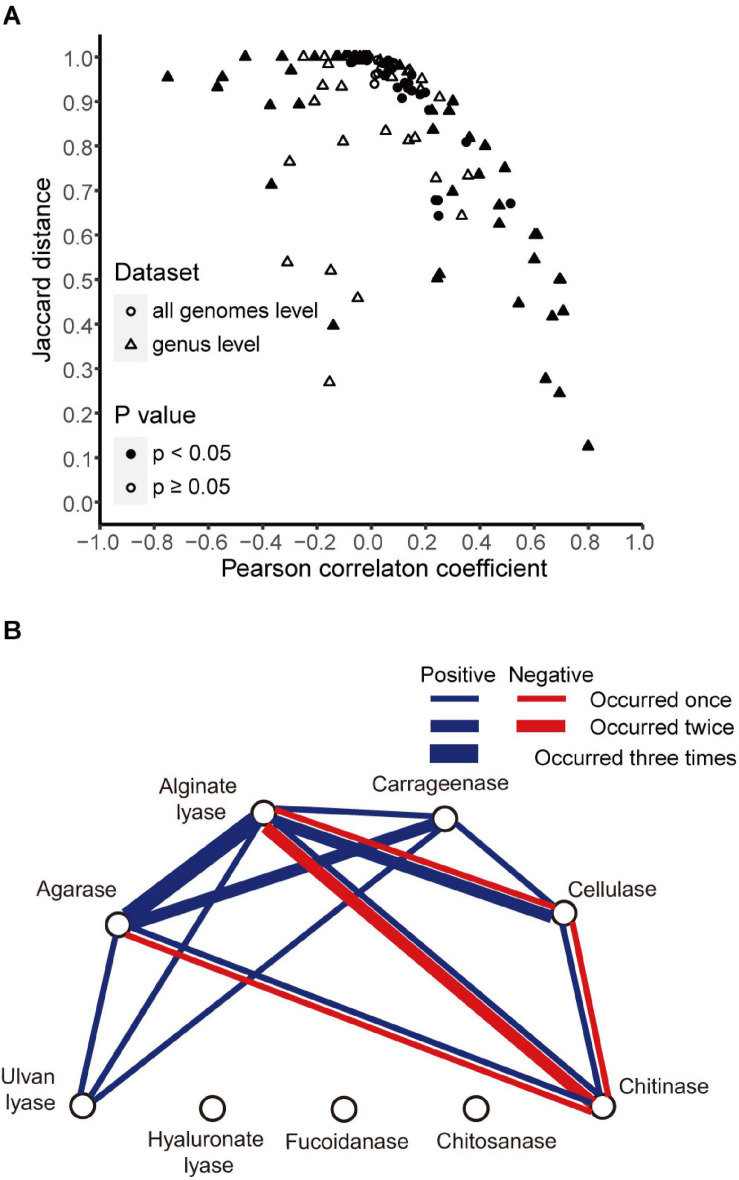
Correlations between enzyme pairs. **(A)** Pearson correlation coefficients and Jaccard distances between enzyme pairs in all genomes (circle) and in 12 genera (triangle). Symbols were filled when *p*-value <0.05 for the Pearson correlation coefficients and open otherwise. **(B)** Correlations between different enzymes found in 12 genera. Blue line, positive correlation; red line, negative correlation. The number of occurrences was indicated with line width (thin, 1; middle, 2; bold, 3).

The above analyses revealed different distributions of different enzymes. Here, we sought to find whether there were correlations between the distributions of different enzymes. To address this issue, we generated for each enzyme a vector containing the presence/absence information in all strains and then calculated Pearson correlation coefficients and Jaccard distances between enzymes. When all genomes in our data set were included, the agarase and carrageenase showed high correlation (*r* = 0.51, *p* < 0.05; Jaccard distance 0.67) (Figure 5A).

Next, the correlations were calculated at the genus level. A total of twelve genera with relatively high numbers of genomes (*n* ≥ 25) were studied. High correlations (|*r*| > 0.4, *p* < 0.05) were detected in five out of the total 12 genera. Among them, all the positive correlations were supported by small Jaccard distances (≤0.75), and all the negative correlations were supported by large Jaccard distances (>0.93) (Figure 5A). A correlation network was constructed accordingly (Figure 5B). A total of six enzymes showed correlations with other enzymes, in one or more genera. Among them, alginate lyases showed the maximal number of correlations with others—they were correlated with all the other five enzymes. The agarases were also widely correlated with the others—they were correlated with four enzymes.

The analyses also revealed negatively correlated enzymes pairs, including alginate lyases–chitinases (in *Pseudoalteromonas* and *Shewanella*), agarases–chitinases (in *Shewanella*), cellulases–chitinases (in *Shewanella*), and alginate lyases–cellulases (in *Pseudomonas*) (Figure 3 for the distribution of these enzymes in the specified genera). It was also noted that for the above four negatively correlated enzyme pairs, both negative and positive correlations were found for each pair (Figure 5B), suggesting different distributions in different genera.

## Discussion

Although the phylogenetic distribution of enzymes degrading polysaccharides cellulose, chitin, and xylan have been studied (Berlemont and Martiny, 2013, 2015), the enzymes degrading polysaccharides from the marine environment have not been systematically studied. The encoding potentials of marine bacteria toward marine polysaccharides remain unclear, hindering our understanding of the ecological roles of different marine bacteria and the ocean carbon cycle. Here, we systematically studied the distribution of nine types of enzymes toward polysaccharides present in marine environments. Compared with the reference database, our annotation revealed a large number of PDE-containing taxa that were not included in the reference database, greatly expanding the known distribution of all nine enzymes at nearly all taxonomic levels. The manually curated reference database together with the stringent annotation pipeline allowed reliable predictions. Therefore, these predicted enzymes may also be used as the reference sequences in the future (meta-)genomic studies.

**FIGURE 6:**
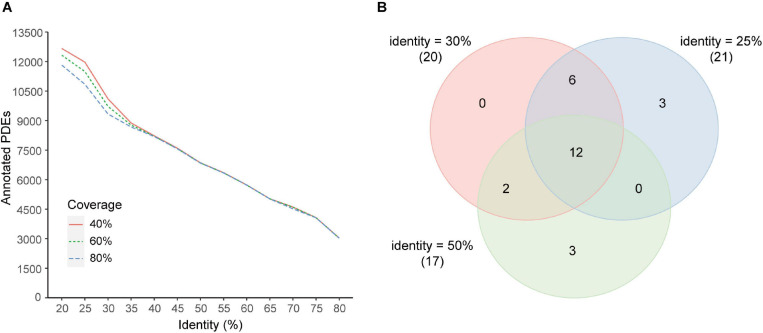
Effects of coverage and identity threshold choice on the results. **(A)** Number of predicted PDEs under different thresholds. **(B)** Venn diagram of correlated enzyme pairs predicted with different thresholds (main set, identity 30%; additional set 1, identity 25%; additional set 2, identity 50%; coverage 80% for all sets). Enzyme pairs with the Pearson correlation coefficient |*r*| > 0.4 and *p* < 0.05 were regarded as correlated. The number of correlated enzyme pairs for each data set was indicated in parentheses beside the set identity.

Different coverage and identity thresholds in the annotation pipeline were tested. It was demonstrated that the coverage threshold had few effects on the number of predicted PDEs with the identity threshold >35% and had minor effects with the identity threshold <35% (Figure 6A). The identity threshold showed moderate effects. For example, when a more stringent identity threshold (50%) was used, predicted PDEs were decreased to about 70% of those obtained with the 30% identity threshold. When a less stringent identity threshold 25% was used, predicted PDEs were increased by about 17%. To further estimate the effects of these parameters on the correlation analyses, correlations between enzymes at the genus level were investigated with two additional data sets (additional set 1: identity 25% + coverage 80%, additional set 2: identity 50% + coverage 80%). Comparison with the main data set revealed that, all the 20 correlated enzyme pairs found with the main data set (identity 30% + coverage 80%) were supported by at least one additional data set, and 12 of them (60%) were found with both additional sets (Figure 6B).

Our analyses revealed different distributions for different enzymes. Cellulases were the most widely distributed among the enzymes studied. This is consistent with that cellulose is the most abundant polysaccharide in nature and is produced by various marine micro-algae and macro-algae (John et al., 2011). Chitinases and alginate lyases were also widely distributed. Consistently, chitin is the second most abundant structural polysaccharide and is produced by arthropods (e.g., crustaceans and insects) and fungi (Rinaudo, 2006). The wide distribution of alginate lyase suggests that alginate is an important carbon resource for marine bacteria. As a matter of fact, a large number of alginate lyases have been characterized from marine bacteria [e.g., see review (Xu et al., 2018), also see Supplementary Table S2 for the reference sequences]. It is unclear why enzymes toward other polysaccharides, including those (agar and carageen) from red algae, are more narrowly distributed.

Our results revealed diversity in the encoding abilities for PDEs in taxa at all levels from phylum to genus. Analysis using consenTRAIT (Martiny et al., 2013) revealed small phylogenetic depths (τ_*D*_) for PDEs, which corresponded to clusters of 16S rRNA identities from 94.4 to 97.1%. The taxonomic threshold based on the 16S rRNA identity is around 96.4% for a bacterial genus (Yarza et al., 2014). Thus, the annotated PDEs were mainly conservatively present at the genus level. Among the enzymes studied (except fucoidanase), the chitinase was the most phylogenetically conserved trait with a τ_*D*_ of 0.0281 for 16S rRNA gene tree and 0.0444 for core gene tree. Consistently, it had the largest mean clade size (4.61 and 5.94 genomes per clade for the 16S rRNA and core gene tree, respectively). It was noted that this depth value was much higher than that reported previously (0.008 for 16S rRNA gene tree) (Zimmerman et al., 2013). The increased depth might be a result of the increased densities of trait-positive genomes on the tree in our data set. Similarly, the mean trait depth for cellulases was also higher than reported previously (0.0226 vs. 0.013 for 16S rRNA gene tree) (Berlemont and Martiny, 2013), which might also be a result of difference in genome data set as well as the gene families used in the analyses.

Our analyses revealed positive correlations between agarases and carrageenases, consistent with that their substrates are both from red algae. Another possibility is that a few bacteria may enrich enzymes for different polysaccharides to enhance their abilities to obtain nutrients from the environment (David et al., 2014; Martiny et al., 2015). Similarly, positive correlations were also observed between alkaline phosphatases, chitinases and β-N-acetyl-glucosaminidases (Zimmerman et al., 2013). Furthermore, our analyses also revealed additional positive correlations when the analyses were limited in a genus, suggesting genetic differences between genera.

Negative correlations have not been reported before. Here our analyses revealed negative correlations at the genus level. The negative correlation was unexpected, because it means that the genus prefers only one type of PDE encoded in one genome. Such distribution patterns suggested physiological and probably ecological differentiation within the genus. The most frequently observed enzyme involved in the negative correlation was the chitinase. It showed negative correlations with the cellulase, alginate lyase, and agarase (Figure 5B). The negative correlation between the chitinase and the other three enzymes seems not a result of the possible incompatibility of the degradation and utilization pathways between chitin and other polysaccharides, since there were indeed strains containing genes for both chitinases and other PDEs. For example, in the genus *Pseudoalteromonas* with *r* = −0.75 for the chitinases-alginate lyases pair, four out of the total 94 genomes encoded both chitinases and alginate lyases. The chitinase substrate, chitin, is rich in the exoskeleton of marine animals like crustaceans, as well as fungal cell wall (Younes and Rinaudo, 2015). Substrates of other enzymes are produced by marine micro- and macro-algae in the marine environment (Michel et al., 2006; Konasani et al., 2018). The negative correlation suggested that one genus was differentiated into two distinct groups (Hehemann et al., 2016; Paulsen et al., 2019), one utilizing the animal-derived polysaccharide chitin and the other utilizing the alga-derived polysaccharides. Since animals and algae represent two distinct habitats for bacteria, spatial separation may allow the bacteria that have been adapted to one type of polysaccharide to lose degrading enzymes toward other polysaccharides.

## Data Availability Statement

The datasets presented in this study can be found in online repositories. The names of the repository/repositories and accession number(s) can be found in the article/Supplementary Material.

## Author Contributions

B-BX and Z-ZS designed the research and wrote the manuscript. B-BX directed the research. Z-ZS performed the experiments and analyzed the data. All authors contributed to the editing and revision of the manuscript, and read and approved the final manuscript.

## Conflict of Interest

The authors declare that the research was conducted in the absence of any commercial or financial relationships that could be construed as a potential conflict of interest.

## Supplementary Material

Supplementary Table 1 Marine-related word list for BioSample filtering.

Supplementary Table 2 Selected reference sequences.

Supplementary Table 3 Marine bacterial genome data set.

Supplementary Table 4 Annotated PDE sequences.
